# Tunable band gap and enhanced thermoelectric performance of tetragonal Germanene under bias voltage and chemical doping

**DOI:** 10.1038/s41598-023-39318-9

**Published:** 2023-07-25

**Authors:** Raad Chegel

**Affiliations:** Department of Physics, Faculty of Science, Malayer University, Malayer, Iran

**Keywords:** Electronic properties and materials, Electronic properties and materials, Two-dimensional materials

## Abstract

This paper employs the tight-binding model to investigate the thermal properties of tetragonal Germanene (T-Ge) affected by external fields and doping. T-Ge is a two-dimensional material with unique electronic properties, including zero band gap and two Dirac points. The electronic properties of T-Ge can be influenced by bias voltage, which can open its band gap and convert it to a semiconductor due to its buckling structure. The tunable band gap of biased T-Ge, makes it a a promising option for electronic and optoelectronic devices. The band structure of T-Ge is split by the magnetic field, leading to an increases its band edges due to the Zeeman Effect. The findings demonstrate that the thermoelectric properties of T-Ge are highly sensitive to external parameters and modifications of the band structure. The thermal and electrical conductivity of T-Ge increase with increasing temperature due to the rise in thermal energy of charge carriers. The thermoelectric properties of T-Ge decrease with bias voltage due to band gap opening, increase with the magnetic field due to a modifications of the band structure, and increase with chemical potential due to increasing density of charge carriers. By manipulating the band structure of T-Ge through bias voltage and chemical doping, the electrical conductivity can be optimized to achieve higher figure of merit (ZT) and improved thermoelectric performance. The results demonstrate the potential of T-Ge for use in electronic and magnetic devices, opening up new possibilities for further research and development in this field.

## Introduction

Two-dimensional (2D) materials, which consist of a single layer or a few layers of atoms, have a wide range of potential applications in future electronics and energy storage devices due to their unique electronic and optical properties. In this regard, numerous studies have been investigated the applications of different well-known nano-sheet structures such as Graphene, BN, SiC, GeC, Germanene and Silicene, in various fields of science and engineering^1–14^.

One of the first and most famous structures from these family structures is Graphene which has a hexagonal structure with two carbon atoms in the unit cell. Graphene has been widely used in various fields including sensors, batteries, and transistors^15^. In the Graphene, the valence and conduction bands meet each other at a single point known as the Dirac point, resulting in several distinct electronic properties such as zero band gap with linear dispersion relations^16^. These features play a crucial role in electronic properties of Graphene and make it an excellent candidate for optoelectronics applications^17^.

Aside from graphene, there are a number of 2D materials such as Germanene and Silicene, that exhibit zero band gap and linear dispersion, similar to graphene. These materials have also emerged as attractive candidates for applications in future technologies due to their intrinsic electronic properties^18^. Germanene is a single layer of germanium atoms arranged in a buckled honeycomb lattice which shows massless Dirac fermions and a high electron mobility due to its zero band gap at the Dirac points^11,12^. These structures exhibit a tunable band gap in the presence of a bias voltage, due to their buckling parameters and their band gap can increased with higher bias strength^19^.

Hexagonal graphene is not the only structure that exhibits gapless Dirac cone features. In addition, the new allotrope of graphene with tetragonal structure which called T-Graphene, is also a metal with two Dirac points in its band structure^20,21^. Note that unlike conventional graphene, which has a hexagonal lattice structure, tetragonal graphene has a square lattice structure^22^. T-Graphene, shows interesting properties which are useful for electronic device applications. For example, at low concentration levels, the substitution of transition metal (TM) can tune the Fermi level and induce magnetic moments in the T-graphene network^21^. Also, the magnitude of the magnetic moment increases as the atomic weight of the dopant atom increases^23^. Also, the non-toxic boron (B)-nitrogen (N) pair substitution on tetragonal graphene (TG) nanodots can be useful for CO gas sensing^24^.

In addition to T-Graphene, materials with the same tetragonal structure, such as T-Germanene (T-Ge), T-Silicene (T-Si) and T-SiC have been predicted and investigated using DFT calculations^25,26^. T-Ge and T-Si are 2D materials composed of a single layer of Ge/Si atoms arranged in a squared lattice structure and they have two Dirac cones in their electronic band structures. The electronic and mechanical properties of these materials have been explored, revealing their potential use in electronic and optoelectronic applications. T-Silicene has two Dirac points in its band structure, resulting in a zero band gap which can be tuned with the application of an electric field and it exhibits elastic properties that are comparable to those of Silicene^27^. In addition, the T-Si has an anisotropic optical response and when it is converted into nanoribbons, its optical properties and thermoelectric properties can be modified by varying the edge width and geometry^26^. The stability and electronic properties of T-SiC are dependent on the arrangement of its constituent atoms and it exhibits a range of electronic properties, including semiconductor, semi-metal, and metal behavior due to the arrangement of their atoms^28^. The predicted properties of T-SiC suggest that it could be highly valuable in a variety of applications, including solar cells, nano-devices, transistors, and phototransistors^29^. The optimized structure for the T-Ge exhibits buckled geometry and it shows interesting properties such as double Dirac points, zero band gap and better thermoelectric behavior than graphene^18^. Due to the buckling structure, the T-Ge structure shows tunable electronic properties with bias voltage and its band gap increases linearly with the strength of the bias voltage^30^.

The thermoelectric properties of 2D materials can be tuned through various methods, such as chemical doping, strain engineering, and surface modification. Optimizing these methods can lead to the development of 2D materials with high ZT values, which can be used to create more efficient and cost-effective thermoelectric devices for a variety of applications including power generation, refrigeration, and thermal management in electronic devices.

Modifying the thermoelectric properties of 2D Germanene structures is essential for the development of more efficient thermoelectric devices that can be used for energy conversion and waste heat recovery^31^. Thermal conductivity in 2D Germanene increases with temperature and is influenced by the bias voltage^32^. Strain engineering can modify the thermal properties of Germanene and the highest power factor for p-type and n-type doping was achieved in hydrogenated monolayer Germanene under biaxial strain of + 3% and − 6%, respectively^33^.

The thermal conductivity in monolayer Germanene is influenced by magnetic fields, with an increase in thermal conductivity as magnetic field strength increases at a fixed temperature^34^. The efficiency of thermoelectric devices is determined by a dimensionless figure of merit, ZT, which depends on the electrical conductivity, thermal conductivity, and Seebeck coefficient. Germanene exhibits a high ZT value at room temperature, making it a promising candidate for thermoelectric applications^35^.

In addition to Germanene, the thermal properties of T-Ge have also been studied. It has been shown that the thermoelectric figure of merit and power of T-Ge increase increase with electric field strength, reaching a maximum value of 0.625 V Å^− 1^^36^. The thermoelectric properties of T-Ge are dependent on the chemical potential and its electrical conductivity and power factor exhibit increases with both positive and negative values of µ^30^.

Until now, unlike intense studies on the mechanical and electronic properties of Germanene, a limited amount of research has been devoted to investigate the thermal properties of T-Ge. This study uses a tight binding model based on previous DFT calculations to investigate the effects of electric and magnetic fields on the properties of T-Ge.

## Model and formalism

### Electronic structure

**Figure 1:**
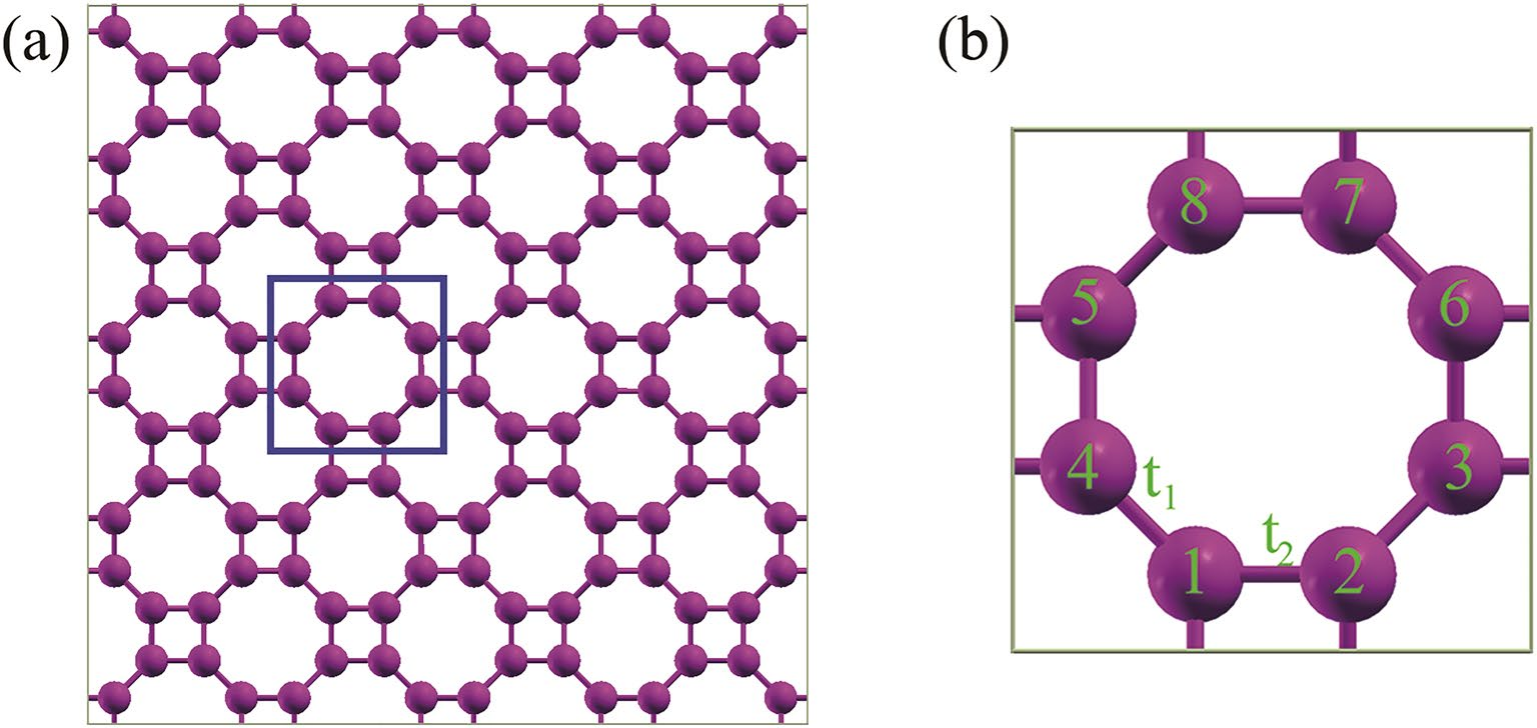
(**a**) Schematic picture of the atomic structure for the T-Ge. The unit cell is shown with black line. (**b**) The unit cell of T-Ge structure with two hopping parameters and eight Ge atoms.

In this section, the Hamiltonian (H) matrix is obtained using the nearest neighbor tight binding model. This model considers the hopping of electrons between nearest-neighbor atoms in the lattice to derive the Hamiltonian matrix elements. In the T-Ge, with eight Ge atoms in the primitive unit cell, the tight binding Hamiltonian requires two hopping parameters, t1 and t2. As shown in the Fig. 1, these parameters represent the hopping energy between neighboring atoms in different directions.

The Hamiltonian matrix for unbiased T-Ge in the absence of a magnetic field can be expressed in second quantization as:

1 $${\text{H}}_{0} = \varepsilon_{0} \mathop \sum \limits_{i} c_{i}^{\dag } c_{i} + t_{1} \mathop \sum \limits_{{\left\langle {i,j} \right\rangle }} \left( {c_{1,i}^{\dag } c_{4,j} + c_{2,i}^{\dag } c_{3,j} + c_{7,i}^{\dag } c_{6,j} + c_{8,i}^{\dag } c_{5,j} + h.c.} \right) + t_{2} \mathop \sum \limits_{{\left\langle {i,j} \right\rangle }} [\left( {c_{1,i}^{\dag } c_{2} + c_{2,i}^{\dag } c_{7,j} + c_{7,i}^{\dag } c_{8,j} + c_{8,i}^{\dag } c_{1,j} + h.c.} \right){ } + \left( {c_{3,i}^{\dag } c_{4,j} + c_{4,i}^{\dag } c_{5,j} + c_{5,i}^{\dag } c_{6,j} + c_{6,i}^{\dag } c_{3,j} + h.c.} \right)]$$

where ${\mathrm{\varepsilon }}_{0}$ is the on-site energy, and $c_{i}^{\dag }$ and $c_{i}$ are the creation and annihilation operators, respectively, for electrons at the i-th lattice site. The summation over i extends over all the lattice sites in the T-Ge structure. The Hamiltonian ${\mathrm{H}}_{0}$ describes the energy of the electrons in the system without the effects of an external fields or other perturbations. However, to include the effects of these perturbations, additional terms may need to be added to the Hamiltonian matrix. Therefore, in the presence of an external electric or magnetic fields, the Hamiltonian matrix needs to be modified.

Based on creation vector ${\Psi }^{\dag } \left( {\varvec{k}} \right) = [c_{1} ,c_{2} ,c_{3} ,c_{4} ,c_{5} ,c_{6} ,c_{7} ,c_{8} ]^{\dag }$ and the $H_{0} = \smallint d\user2{k }{\Psi }^{\dag } \left( {\varvec{k}} \right) H_{0} \left( {\varvec{k}} \right) {\Psi }\left( {\varvec{k}} \right)$, the $H_{0} \left( {\varvec{k}} \right)$ can be written as:

2 $$H\left({\varvec{k}}\right)=\left(\begin{array}{cc}{H}_{11}& {H}_{12}\\ {H}_{21}& {H}_{22}\end{array}\right)$$

$$H_{11} \left( {\varvec{k}} \right) = \left( {\begin{array}{*{20}c} 0 & {t_{2} } & 0 & {t_{1} } \\ {t_{2} } & 0 & {t_{1} } & 0 \\ 0 & {t_{1} } & 0 & {f_{1} } \\ {t_{1} } & 0 & {f_{1}^{\dag } } & 0 \\ \end{array} } \right) \;\;\;\; H_{22} \left( {\varvec{k}} \right) = \left( {\begin{array}{*{20}c} 0 & {f_{1}^{\dag } } & 0 & {t_{1} } \\ {f_{1} } & 0 & {t_{1} } & 0 \\ 0 & {t_{1} } & 0 & {t_{2} } \\ {t_{1} } & 0 & {t_{2} } & 0 \\ \end{array} } \right)\;\;\;\;\;H_{21} \left( {\varvec{k}} \right) = \left( {\begin{array}{*{20}c} 0 & 0 & 0 & {f_{2}^{\dag } } \\ 0 & 0 & {f_{2}^{\dag } } & 0 \\ 0 & {t_{2} } & 0 & 0 \\ {t_{2} } & 0 & 0 & 0 \\ \end{array} } \right)$$

where ${f}_{1}={t}_{2}{e}^{i{k}_{x}a}$ and ${f}_{2}={t}_{2}{e}^{i{k}_{y}a}$ and ${\varvec{k}}={k}_{x} {\widehat{e}}_{x}+{k}_{y} {\widehat{e}}_{y}$ is the wave vector in the first Brillouin zone. The band structure $E({\varvec{k}})$ of unbiased T-Ge structures can be obtained by diagonalizing the overall Hamiltonian matrix, using on the wave vector ${\varvec{k}}$ in the first Brillouin zone. The hopping integral ${t}_{i}$ are given in Ref.^30^, based on the DFT calculations.

Monolayer T-Ge has a non-zero buckling parameter in its stable form. However, when it is subjected to a vertical electric field F, the Hamiltonian $H_{U} = \mathop \sum \limits_{i} U_{i} c_{i}^{\dag } c_{i}$ is added, causing its electronic structure to become dependent on the strength of the electric field. Note that, in the presence of vertical electric field **F**, the bias voltage strength $U_{i} = \pm {\varvec{F}}.\frac{{\Delta {\varvec{z}}}}{2}$ appears to the top and bottom Ge atoms of the buckled T-Ge. On the other hand, when a magnetic field ${\Pi }$ $\left[ {{\Pi }\left( {B_{0} } \right) = \sigma g\mu_{B} B_{0} } \right]$ is applied to the trilayer system, the overall Hamiltonian becomes spin-dependent and can be represented by a 16 × 16 matrix. By solving the Schrödinger equation, the band structure $E^{\sigma } \left( {{\varvec{k}},U,{\Pi }} \right)$ can be obtained, where σ represents the spin index. The band structure can provide insight into the effects of the bias voltage U and magnetic field Π on the electronic properties of T-Ge, such as its thermal and electrical conductivity.

### Thermal properties

The Green function ${\varvec{G}}\left(E\right)$ can be calculated by solving the electrons’ equation of motion^37^:

3 $$\sum_{s}\left[\left(E{\varvec{I}}-{\varvec{\Delta}}(\mathbf{F})\right){\delta }_{is}+{{\varvec{t}}}_{is}\right]{{\varvec{G}}}_{sj}\left(E\right)={\varvec{I}}{\delta }_{ij}$$

The density of state (DOS) can then be found with $-{\frac{1}{{\varvec{\pi}}}{\varvec{I}}{\varvec{m}}[{\varvec{G}}}_{jj}\left(E\right)]$. The delta Kronecker, hopping integral, as well as on site potential matrixes are represented by $\delta$, ${\varvec{t}}$ and ${\varvec{\Delta}}(\mathbf{F})$, respectively.

In this study, the Kubo-Greenwood formula was used to investigate the temperature dependence of the thermal characteristics of the T-Ge structure. The spectral function ${\varvec{A}}\left({\varvec{k}},\varepsilon \right)=-2Im\widehat{G}\left(k,\varepsilon \right)$, associated with the DOS and transport coefficients ${\Lambda }_{mn}$, was evaluated using the Green's function.

When a temperature gradient ∇T is present, the electrical charge current ${J}^{e}$ and thermal heat current ${J}^{Q}$ are defined in terms of the transport coefficients ${\Lambda }_{q{q}{\prime}}$, as shown in equation^38^:

4 $$\left(\begin{array}{c}{\widehat{J}}_{1}\equiv {\widehat{J}}^{e}\\ {\widehat{J}}_{2}\equiv {\widehat{J}}^{Q}\end{array}\right)=\left(\begin{array}{cc}{\Lambda }_{11}& {\Lambda }_{12}\\ {\Lambda }_{21}& {\Lambda }_{22}\end{array}\right)\left(\begin{array}{c}E\\ \nabla \mathrm{T}\end{array}\right)$$

The ${\Lambda }_{mn}$ coefficients were obtained in terms of the correlation function among the charge and heat current operators as^39^:

5 $${\Lambda }_{q{q}{\prime}}=\frac{i}{\beta \omega } \underset{i{\omega }_{n}\to \omega +i{0}^{+}}{\mathrm{lim}}{\int }_{0}^{\beta }d\tau {e}^{i{\omega }_{n}\tau } \langle {T}_{\tau } {\widehat{J}}_{q}\left(\tau \right){\widehat{J}}_{{q}{\prime}}\left(0\right)\rangle$$

The charge current operator function ${\widehat{J}}^{e}$ and the heat current operator ${\widehat{J}}^{Q}$ can be defined as^38^

6a $$\hat{J}^{e} = \mathop \sum \limits_{k} \mathop \sum \limits_{p} \nu_{k}^{\left( p \right)} c_{k}^{\dag \left( p \right)} c_{k}^{\left( p \right)}$$

6b $$\hat{J}^{Q} = \mathop \sum \limits_{k} \mathop \sum \limits_{p} E_{p} \left( {{\varvec{k}},{\Pi }} \right) \nu_{k}^{\left( p \right)} c_{k}^{\dag \left( p \right)} c_{k}^{\left( p \right)}$$

Here, ${\nu }_{k}^{(p)}={\partial }_{k}{E}_{p}({\varvec{k}},U,\Pi )$ represents the velocity operator assigned for the p-th Hamiltonian eigenvalues in the presence of an external fields.

By using the Wick’s theorem associated with the Green function $G_{p} \left( {k,\tau } \right) = - \left\langle {T_{\tau } { }c_{k}^{\dag \left( p \right)} \left( \tau \right){ }c_{k}^{\left( p \right)} \left( 0 \right)} \right\rangle$, the correlation function components can be written as:

7 $$\langle {T}_{\tau } {\widehat{J}}_{q}\left(\tau \right){\widehat{J}}_{{q}{\prime}}\left(0\right)\rangle =\sum_{k,p}{\left({E}_{p}\left(k,U,\Pi \right)\right)}^{q+{q}{\prime}-2}{\left({ \nu }_{k}^{\left(p\right)}\right)}^{2}{G}_{p}(k,\tau ){G}_{p}(k,-\tau )$$

where ${G}_{p}(k,\tau )$ is the Green function, ${T}_{\tau }$ is the time-ordering operator, ${E}_{p}\left(k,U,\Pi \right)$ is the energy of an electron with momentum k in the presence of a bias voltage U and magnetic field.

Utilizing the Fourier transform of Green function [${G}_{p}\left(k,\tau \right)=\sum_{m}{e}^{-i{\omega }_{m}\tau }{G}_{p}\left(k,i{\omega }_{m}\right)$], the transport coefficients Eq. (5) may be expressed as:

8 $${\Lambda }_{\mathrm{q}{q}{\prime}}=\frac{i}{{\beta }^{2}\omega } \underset{i{\omega }_{n}\to \omega +i{0}^{+}}{\mathrm{lim}} \sum_{k,p}\sum_{m}{\left({E}_{p}\left(k,U,\Pi \right)\right)}^{\mathrm{q}+{q}{\prime}-2}{\left({ \nu }_{k}^{\left(p\right)}\right)}^{2} {G}_{p}(k,i{\omega }_{m}){G}_{p}(k,i{\omega }_{n}+i{\omega }_{m})$$

which the ${G}_{p}\left(k,i{\omega }_{m}\right)={\int }_{-\infty }^{+\infty }\frac{d\varepsilon }{2\pi } \frac{{A}_{p}\left(k,\varepsilon \right)}{i{\omega }_{m}-\varepsilon }$ is associated with the spectral function ${A}_{p}\left(k,\varepsilon \right)$. Finally, by utilizing the Matsubara frequency summation along with Eq. (8), the transport coefficient ${\Lambda }_{mn}$ obtained from the following equation:

9 $${\Lambda }_{\mathrm{q}{q}{\prime}}=\frac{1}{\beta } {\int }_{-\infty }^{+\infty }\left[\frac{\partial f\left(\varepsilon \right)}{\partial \varepsilon }\right]\frac{d\varepsilon }{2\pi } \sum_{k,p}{\left({E}_{p}\left(k,U,\Pi \right)\right)}^{\mathrm{q}+{q}{\prime}-2}{\left({ \nu }_{k}^{\left(p\right)}{A}_{p}\left(k,\varepsilon \right)\right)}^{2}$$

Here, the Fermi-Dirac distribution function is expressed using the $f\left(\varepsilon ,T\right)={[1+exp\left(\varepsilon /{k}_{B}T\right)]}^{-1}$. The electrical conductivity as a function of temperature, $\sigma \left(\mathrm{T}\right)$, is proportional to the transport coefficient ${\Pi }_{11}$. The thermal conductivity as a function of temperature is obtained by the following equation^40^:

10 $$\upkappa \left(T\right)=\frac{{\mathrm{k}}_{B}^{2}}{T}\left[{\Lambda }_{22}\left(T\right)-\frac{{\Lambda }_{12}\left(T\right){\Lambda }_{21}\left(T\right)}{{\Lambda }_{11}\left(T\right)}\right]$$

By using the obtained equations for the electrical and thermal conductivity, the temperature dependence of the thermoelectric figure-of-merit [$ZT=\frac{\sigma {S}^{2}T}{\kappa }$] and thermopower $\mathrm{PF}\left(T\right)$ can be obtained as^40^:

11 $$ZT(\mathrm{T})=\frac{[{\Lambda }_{12}{]}^{2}}{{\Lambda }_{11}{\Lambda }_{22}-[{\Lambda }_{12}{]}^{2}}$$

$$\mathrm{PF}\left(T\right)=\frac{[{\Lambda }_{12}{]}^{2}}{{\mathrm{T}}^{2}{\Lambda }_{11}}$$

The paramagnetic susceptibility are related to the DOS $[D\left(\varepsilon \right)]$ and is obtained by^41^:

12 $$\upchi \left(\mathrm{T}\right)={\int }_{-\infty }^{\infty }\varepsilon \left[\frac{\partial f\left(\varepsilon ,T\right)}{\partial \varepsilon }\right]D\left(\varepsilon \right) d\varepsilon$$

To investigation the thermal conductivity as a function of the chemical potential, the transport coefficients are described with^42^:

13 $${\Lambda }_{\mathrm{ij}}(\mu )=\int d\varepsilon \left[\frac{-\partial f\left(\varepsilon -\mu \right)}{\partial \varepsilon }\right] {(\varepsilon -\mu )}^{j-i}\alpha (\varepsilon )$$

where the $\alpha (\varepsilon )$ is the spectral conductivity.

## Electronic structures

**Figure 2:**
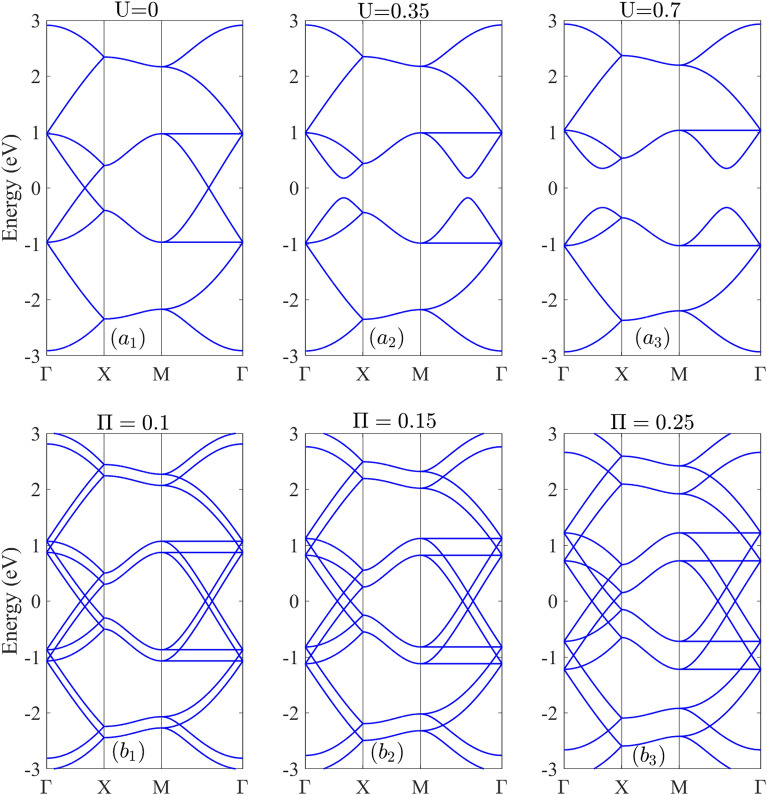
The band structure of the T-Ge (**a1**)–(**a3**) without magnetic field and in the presence of the bias voltages U = 0, 0.35 and 0.7 eV and (**b1**)–(**b3**) without bias voltage and in the presence of the magnetic fields Π = 0.1, 0.15 and 0.25, respectively.

Figure 2 illustrates the band structure of T-Ge when subjected to a bias voltage U and magnetic field Π. The band structure exhibits several valence and conduction subbands on both sides of the Fermi level. Without external fields, T-Ge is metallic because the lowest and highest bands touch at the Fermi level, and there are double Dirac points present in the ΓX and MΓ directions. The band dispersion around these points is linear with respect to the wave vector [Fig. 2a1]. In the presence of a bias voltage U = 0.3 eV, the band gap opens at both Dirac points, and the band structure exhibits a parabolic behavior around these points [Fig. 2a2]. As the bias voltage strength increases to U = 0.7 eV, the vertical distance between the valence and conduction bands at the Dirac points increases, leading to an increase in the band gap. These findings are consistent with previous studies of T-Si with different electric field strengths^26^, and they agree with the findings of Ref.^30^ for T-Ge.

As shown in Fig. 2b1, b2 and b3, when a magnetic field Π = 0.1, 0.15 and 0.25 is applied to T-Ge, the magnetic moments of the electrons in the structure align with the field, causing a splitting of each valence and conduction band into two subbands with spin up and down. This feature is known as the Zeeman Effect. At any magnetic field strength, the highest and lowest subbands touch at four points on the Fermi level, resulting in a zero band gap. As a result of applying the magnetic field, the band edges and flatting the subbands change with the magnetic field strength Π. in the MΓ direction, the subbands become flattened in energy at around E =  ± 1 eV. As the magnetic field strength increases, the distance between neighboring subbands also increases. Monolayer phosphorene exhibits a similar pattern of splitting the valence and conduction subbands due to the presence of a Zeeman magnetic field^43^.

As shown in Fig. S1, when a magnetic field Π = 0.1, 0.2 and 0.3 is applied to a biased T-Ge with U = 0.7 eV, each energy level splits into two sublevels due to the Zeeman Effect. The splitting distance between the sublevels increases with increasing Π. Additionally, the non-zero band gap of biased T-Ge with U = 0.7 eV decreases with magnetic field until it vanishes at a critical value of Π = 0.35.

It can be concluded that the bias voltage and magnetic field have opposing effects on the electronic structure of T-Ge. The bias voltage increases the band gap and decreases electronic transport, while the magnetic field has a contrasting effect, decreasing the band gap and enhancing the electronic transport. Understanding the sensitivity of the electronic properties of T-Ge to external fields is important for design and optimizing of electronic devices based on T-Ge, such as transistors and sensors.

## Effects of the temperature on thermal properties

### Thermal conductivity

**Figure 3:**
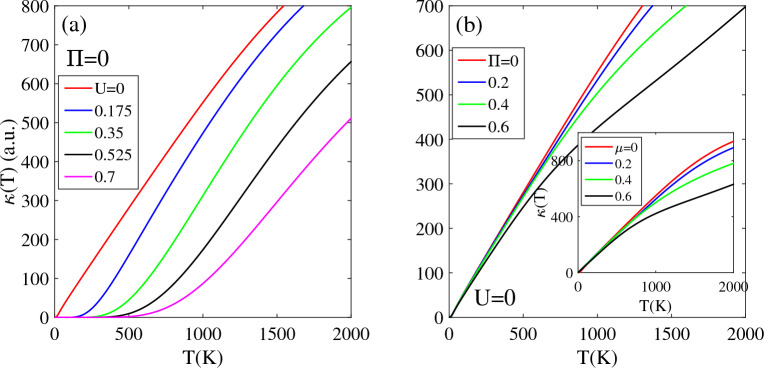
The thermal conductivity of the T-Ge structure in terms of the temperature in the presence of the (**a**) bias voltage with Π = 0 and (**b**) magnetic field without bias voltage. Inset (**b**) shows effects of the chemical potential µ on the κ(T) without bias voltage.

Figure 3a shows thermal conductivity κ(T) for undoped T-Ge with different values of bias voltages, in the absence of the magnetic field. In the unbiased case, the κ(T) value is zero at absolute temperature T = 0 K, because there are no free carriers available for heat transfer. However, as the temperature increases, the κ(T) of the T-Ge exhibits a linear increase due to the excitation of charge carriers to higher energy levels.

In the case of a biased T-Ge with a non-zero band gap, the rate of increase in κ(T) depends on the magnitude of the bias voltage U. This is because the non-zero band gap acts as a potential barrier for the excitation of charge carriers. For the biased T-Ge, at low temperatures, there exists a temperature range called the "TZ region" where κ(T) remains zero due to the band gap opening. In this region, the thermal energy is insufficient to excite electrons from the valence to the conduction bands. The width of the TZ region depends on the magnitude of the bias voltage U, and it is about 150 K for U = 0.175 eV and increases to 500 K for U = 0.7 eV. However, as the temperature continues to increases beyond the TZ region, the κ(T) begins to increase and becomes non-zero because the charge carriers obtain enough energy to transition to higher energy levels. The strength of the bias voltage also affects the behavior of κ(T) in T-Ge. A stronger electric field leads to a smaller κ(T), because the thermal transition rate of electrons decreases due to the increasing band gap and fewer electrons are available to conduct to higher energy levels. It is worth noting that, similar to T-Ge, monolayer Germanene exhibits a similar trend for κ(T) with respect to temperature when a bias voltage is applied^32^.

One interesting physical feature of T-Ge is the effect of Π on its κ(T) without bias, as demonstrated in Fig. 3b. The presence of magnetic fields can affect the κ(T) of T-Ge by modifying energy dispersion relation, DOS and effective mass of the carriers. This, in turn, can affect the transport properties of the T-Ge, including the thermal conductivity.

In the presence of a non-zero magnetic field (Π ≠ 0), unbiased T-Ge exhibits a zero-band gap, meaning that there are no energy bands separating the highest valence and lowest conduction subbands. As a result, even with the application of low thermal energy, the charges can be easily excited to higher energy levels. Therefore, in the presence of Π ≠ 0, the κ(T) does not approach zero within any temperature region below 2000 K, and a TZ region does not exist. This is in contrast to the effects of bias voltage, which can cause κ(T) to reach zero in low temperature region. This unique behavior of κ(T) in Π ≠ 0 is consistent for different magnetic field strengths, as shown in Fig. 3b. At higher temperatures, the intensity of κ(T) is smaller for stronger magnetic fields Π compared to weaker Π, and the κ(T, Π = 0) is larger than other selected Π.

This phenomenon can be explained by the charge excitation rate under the influence of a magnetic field. A stronger Π strength leads to movement of a greater number of charge carriers to higher energy levels, thereby increasing the population of excited charges in upper levels. The increased scattering of charge carriers can affect the transport properties and decrease the κ(T). The findings show that κ(T) increases with temperature in any Π, and it has a larger magnitude for weaker Π than stronger Π. The obtained results are consistent with the work done on the Silicene monolayer^44^.

Similar to the influence of Π, the thermal conductivity of unbiased T-Ge is also affected by chemical potential parameter µ. As shown in the inset of Fig. 3b, κ(T) exhibits an increasing behavior with varying chemical potential µ. Due to the zero band gap for unbiased T-Ge in Π = 0, κ(T, µ) doesn't reach zero and there is no zero temperature region TZ below 2000 K. As the temperature increases, κ(T, µ) increases linearly, but the rate of increase with temperature decreases with increasing µ. As shown in inset Fig. 3b, κ(T) is the largest at µ = 0 while it decreases at non-zero values of µ. This behavior can be attributed to the changes in the concentration of excited charge carriers and their scattering mechanisms with increasing µ. This is because at higher µ values, the concentration of excited charge carriers in upper levels increases, leading to an increase in scattering and a decrease in the mobility and thermal conductivity. For instance, κ(T, µ = 0) is larger than κ(T, µ ≠ 0), and their difference increases with increasing µ.

**Figure 4:**
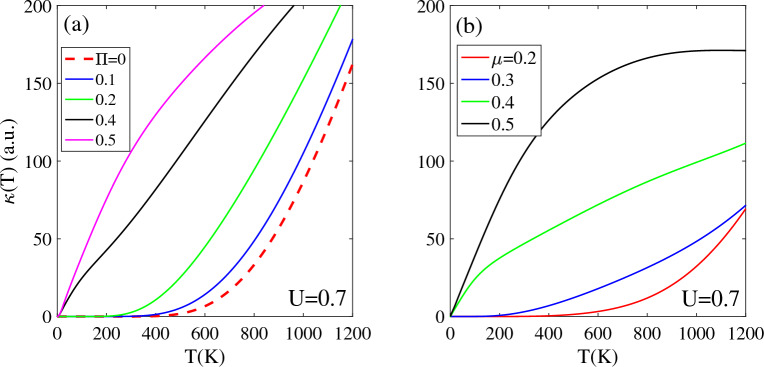
Effects of the (**a**) magnetic field and (**b**) chemical potential on the thermal conductivity of biased T-Ge structure in terms of the temperature.

Figure 4a and b demonstrate that the thermal conductivity of biased T-Ge is affected by both the magnetic field Π and chemical potential µ. Biased T-Ge has a non-zero band gap, which acts as a potential barrier that prevents the excitation of charge carriers from the valence to conduction bands. This results in a zero intensity of κ(T) below TZ = 500 K.

For biased T-Ge with bias voltage strength U = 0.7 eV, applying a magnetic field Π and chemical potential µ significantly increases κ(T) through different mechanisms. The band gap of biased T-Ge decreases when a magnetic field Π is applied, which allows for easier excitation of charge carriers to higher levels and this leads to an increase in κ(T, U = 0.7). As Π increases from 0.1 to 0.2, the TZ region of κ(T, U = 0.7) decreases from 500 to 300 K, and for Π ≥ 0.4, it completely vanishes. The rate of increase of κ(T, U = 0.7,Π ≠ 0) is dependent on magnetic field strength Π. Specifically, κ(T, U = 0.7, Π = 0.4) exhibits a linear increasing with temperature, with a lower intensity compared to κ(T, U = 0.7, Π = 0.5) and the difference between them, decreases at higher temperatures. The κ(T) of biased T-Ge demonstrates an increase with Π at any fixed temperature below 1200 K. Specifically, at the same temperature, the κ(U = 0.7, Π = 0.5) exhibits a significantly larger value compared to other Π values.

Figure 4b shows the effect of chemical potential µ on the temperature dependence of κ(T) for biased T-Ge. The κ(T, U = 0.7) demonstrates an increasing trend with rising temperature for each selected value of µ = 0.2, 0.3, 0.4, and 0.5 eV. This behavior can be attributed to the higher concentration of charge carriers, which enhances the transition rate of electrons between energy levels. For lower values of µ ≤ 0.3, there is a non-zero TZ region where the κ(T, U = 0.7) vanishes due to the non-zero band gap of biased T-Ge. For µ = 0.2 eV, the κ(T, U = 0.7) is zero for temperatures TZ ≤ 420 K, which decreases to TZ ≤ 200 K for µ = 0.3 eV. As µ increases, the width of the TZ region decreases and completely vanishes for µ ≥ 0.4 eV. For chemical potential values of µ ≥ 0.4 eV, the κ(T, U = 0.7) is non-zero for all temperatures above T = 0 K and increases linearly with temperature. At a constant temperature, κ(T, U = 0.7) is larger for higher values of µ compared to smaller values. As shown in Fig. 4b, κ(U = 0.7, µ = 0.5) is larger than other selected values of µ with smaller strengths.

### Electrical conductivity

**Figure 5:**
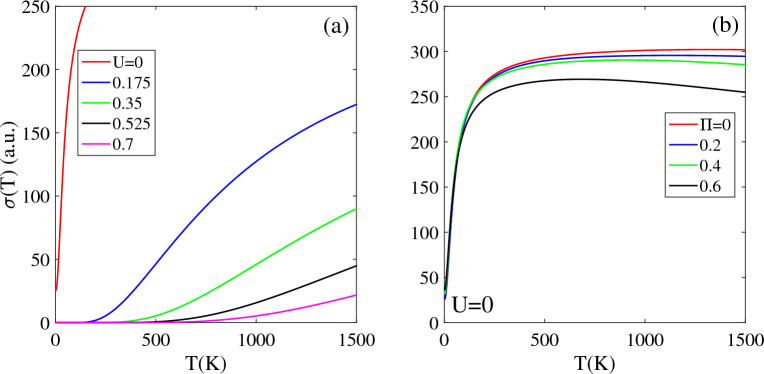
The electrical conductivity of the T-Ge structure in terms of the temperature in the presence of the (**a**) bias voltage with Π = 0 and (**b**) magnetic field without bias voltage.

Figure 5 illustrates the variation of the electrical conductivity σ(T) of undoped T-Ge with temperature, for different bias voltages and magnetic field. The effects of the bias voltage exhibit several interesting features. The σ(T) of unbiased T-Ge increases sharply with temperature above absolute zero, and does not have a zero intensity in the temperature range T < 1500 K, due to its zero band gap.

When a bias voltage of U = 0.175 eV is applied, the σ(T,U) significantly decreases and becomes zero in a low-temperature region TZ. The TZ region depends on the bias voltage and increases with U. Specifically, it is approximately 165 K for U = 0.175 eV, which increases to 330 K and 550 K for U = 0.35 eV and 0.7 eV, respectively. The increasing the electrical conductivity of T-Ge with temperature is consistent with similar trends observed in other materials, such as single-layer Silicene^45^.

The existence and expansion of the TZ region can be explained by the behavior of the band gap of T-Ge under the influence of bias voltage. A stronger bias voltage increases the band gap of T-Ge, which requires more thermal energy to excite the charge carriers. As a result, σ(T, U) remains zero over a broader TZ region. Above the TZ region, σ(T, U) increases as the temperature rises further, but the rate of increase decreases for stronger bias voltage. As shown in Fig. 5a, the intensity of σ(T, U = 0.175) is the highest, while σ(T, U = 0.7) exhibits the lowest intensity, in the entire temperature range of T < 1500 K.

Figure 5b demonstrates the effect of a magnetic field on the electrical conductivity of unbiased T-Ge, under the half-filling constraint (µ = 0). For all selected values of Π, the σ(T, Π ≠ 0) increases from zero with temperature until reaching a maximum value at T≈250 K, and it remains constant above 250 K.

Due to the zero band gap of unbiased T-Ge in the presence of a magnetic field, the σ(T, Π ≠ 0) remains non-zero below T ≤ 1500 K. The electrical conductivity for Π = 0 is higher than that of Π ≠ 0 because: (i) in presence of the magnetic field, all structures remain metallic without a band gap, (ii) a higher magnetic field strength increases the DOS around the Fermi level, and at a constant temperature, more charge carriers can transfer to higher energy levels, and (iii) the increased number of charge carriers at higher energy levels leads to increased carrier scattering, which in turn reduces carrier mobility and ultimately decreases the electrical conductivity. In this case, as depicted in Fig. 5b, the σ(T,Π = 0.6) demonstrates a mirror decreasing pattern with increasing temperature for T > 1000 K. Additionally, the intensity of σ(T, Π = 0.6) is smaller compared to the other selected Π values.

**Figure 6:**
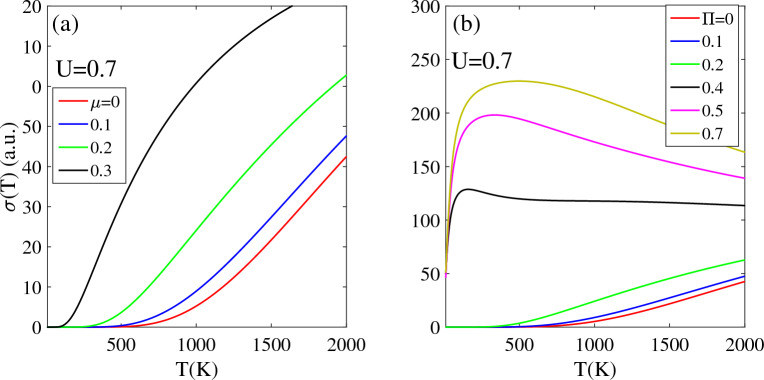
Effects of the (**a**) chemical potential and (**b**) magnetic field on the electrical conductivity of biased T-Ge structure in terms of the temperature.

Figure 6 illustrates the impact of a magnetic field Π and chemical potential µ on the electrical conductivity of biased T-Ge with U = 0.7 eV. Note that, in the presence of the U = 0.7 eV, the T-Ge has a non-zero band gap with TZ = 500 K. As shown in the Fig. 6a, the TZ region is dependent on the chemical potential, and it decreases from 450 K at µ = 0.1 eV to 260 K and 95 K for µ = 0.2 eV and 0.3 eV, respectively. This behavior indicates that at non-zero chemical potential (µ ≠ 0), there are more electrons available to conduct electricity at lower temperatures. Furthermore, above the TZ region, the σ(U, µ) is higher for larger value of µ because the density of charge carriers is directly related to the chemical potential. Notably, for µ = 0.3 eV, the electrical conductivity is significantly enhanced at lower temperatures due to the greater number of available charge carriers. It should be noted that for chemical potentials larger than 0.4 eV, the electrical conductivity σ(U = 0.7, µ > 0.4) remains non-zero in the T < 2000 K range without exhibiting a TZ region.

As shown in Fig. 6b, the electrical conductivity of biased T-Ge with U = 0.7 eV shows a dependence on the applied magnetic field Π. The band gap of biased T-Ge decreases with increasing magnetic field Π strength. This leads to an increase in electrical conductivity from lower temperatures, because the lower band gap enables the easier excitation of charge carriers. When Π increases to 0.2, the σ(T, U = 0.7) remains zero in TZ < 400 K due to the presence of a non-zero band gap at [Π = 0.2 and U = 0.7 eV].

As the magnetic field strength Π exceeds 0.4, the band gap of biased T-Ge eventually vanishes and the electrical conductivity shows a rapid increase with increasing temperature above T = 0 K. Specifically, at Π = 0.4, the σ(T, U = 0.7) increases as temperature is raised to 120 K and then remains approximately constant in T > 120 K. For stronger magnetic field strengths such as Π = 0.5 and 0.6, the electrical conductivity increases with temperature until reaches 200 K and 250 K, respectively. However, beyond these temperatures, there is a slight decrease in the electrical conductivity as temperature is further increased to T = 2000 K.

An interesting observation is that the difference in electrical conductivity between biased T-Ge with smaller [Π ≤ 0.2] and larger [Π ≥ 0.4] magnetic fields decreases at higher temperatures. This can be explained as follows. For lower values of Π (Π ≤ 0.2), the band gap of biased T-Ge decreases as the magnetic field strength increases. This reduction in the band gap allows more charge carriers to transition to higher energy levels, which results in an increase in electrical conductivity. In contrast, for Π ≥ 0.4, the band gap in biased T-Ge completely disappears and remains at zero. This can result in more scattering of charge carriers and ultimately lead to a reduction in electrical conductivity.

After analyzing the data presented in the Fig. 6, it can be concluded that the electrical conductivity of biased T-Ge is influenced by two main factors: the strength of the applied magnetic field [due to the decrease in band gap] and the chemical potential [which leads to a higher density of charge carriers].

### Thermoelectric properties

**Figure 7:**
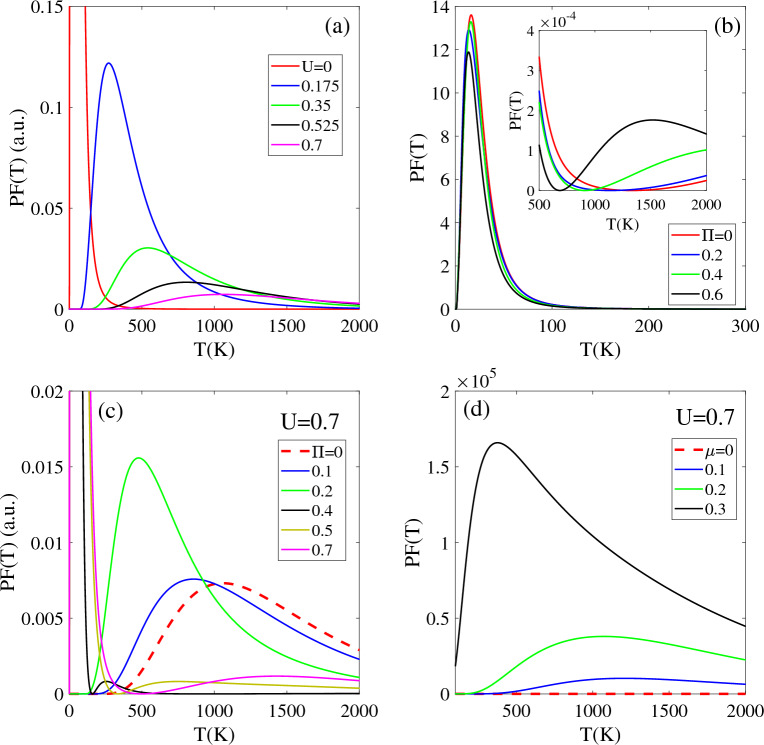
The power factor PF(T) in terms of the temperature in the presence of the (**a**) bias voltage and (**b**) magnetic field. Effects of the (**c**) magnetic field and (**d**) chemical potential on the PF(T) of the biased T-Ge structure.

Figure 7 displays the power factor PF(T) of T-Ge as a function of temperature for different bias voltages, magnetic fields and chemical potentials. The PF(T) of an unbiased T-Ge structure exhibits a pronounced peak at lower temperatures (T < 500 K) and then decreases significantly at higher temperatures (T > 1000 K), as shown in Fig. 7a. The curve also shows that the power factor is strongly dependent on the bias voltage. When a bias voltage U = 0.175 eV is applied, the intensity of the PF exhibits a decrease in intensity compared to the unbiased structure, and a peak appears at 270 K. However, as the bias voltage increased to U = 0.375 eV and 0.525 eV, the peak in the PF(T) shifts to higher temperatures of about 510 K and 780 K, respectively, with a noticeable decrease in intensity. Interestingly, the PF(T) intensity of T-Ge decreases with increasing bias voltage, which can be attributed to the opening and increasing of the band gap in response to the applied bias voltage. At higher temperatures above 1000 K, the difference in intensity between the PF(T) of the unbiased structure and the biased structures decreases,. Moreover, the stronger bias voltage exhibits a higher intensity in the PF(F). It can be concluded that the PF(T) of T-Ge can be strongly influenced by both temperature and bias voltage. A similar increase in PF(T) with temperature is seen in monolayer ZnO^46^ and Si2BN^47^.

Figure 7b depicts the effects of magnetic field Π on the temperature-dependent PF(T) of unbiased T-Ge in low [T < 300 K] and high [T < 2000 K] temperature regions. In all cases, a prominent peak with high intensity is observed at temperatures below 100 K, and this peak occurs at approximately the same temperature. The convergence of the PF(T) intensity towards a similar value at low temperatures suggests that the influence of the magnetic field on the PF(T) intensity becomes less pronounced in the low-temperature range below 300 K. As shown in inset Fig. 7b, a significant change in the behavior of PF(T) occurs for temperatures above approximately 500 K. Unlike in the low-temperature region, the PF(T) exhibits an increasing trend with rising temperature, and stronger magnetic fields lead to higher intensities for PF(T). In comparison to the Fig. 7a, we can observe that the effect of magnetic field Π on PF(T) is different from the effect of bias voltage U. While increasing U generally leads to a decrease in PF(T), the effect of Π is more complex and dependent on the temperature region. Specifically, in the low-temperature region, PF(T) remains unaffected by Π, whereas in the higher temperature region, it exhibits an increasing behavior pattern in response to variations in Π.

Figure 7c, d illustrate the effects of chemical potential and magnetic field on the PF(T) of biased T-Ge with a bias voltage of U = 0.7 eV. Without a magnetic field Π, the PF(T) intensity is zero below a temperature of 500 K, increases to its peak at 1100 K, and then decreases with further increases in temperature. When magnetic field values of Π = 0.1 and 0.2 are applied to PF(U = 0.7), the PF(U,Π) remains zero up to temperatures of 250 K and 150 K, respectively. After reaching these temperatures, the PF(U, Π) exhibits an increasing trend. Additionally, by applying and increasing Π, the peak of PF(U = 0.7,Π) moves to lower temperatures with enhanced intensity. For stronger Π values of 0.5 and 0.7, the intensity of PF(U, Π) significantly increases in T < 500 K. However, above T > 1000 K, the behavior of PF(U = 0.7, Π) in terms of temperature changes and the smaller magnetic field leading to larger intensity for PF(U = 0.7, Π). Furthermore, the difference between all cases decreases, and their PF reaches the same value as the temperature approaches 2000 K. To further clarify, the effects of the chemical potential µ on the biased T-Ge is investigated in Fig. 7d. In this case, when the chemical potential µ is applied to the biased T-Ge, it causes a significant increase in the intensity of PF(T). For µ = 0.2 eV, the PF(T) increases above 280 K until it reaches a peak at 1200 K, and then decreases. The intensity of the PF(T) significantly increases with a stronger chemical potential of µ = 0.3 eV. In this case, the PF(T) becomes nonzero across the entire temperature range up to 2000 K with the peak position at 360 K. Similar to the effects of Π on biased T-Ge, the differences between all values of µ for PF(T) of biased T-Ge decrease for temperatures near 2000 K. These similarities suggest that both Π and µ play important roles in determining the thermoelectric properties of T-Ge, and that their effects are temperature-dependent.

**Figure 8:**
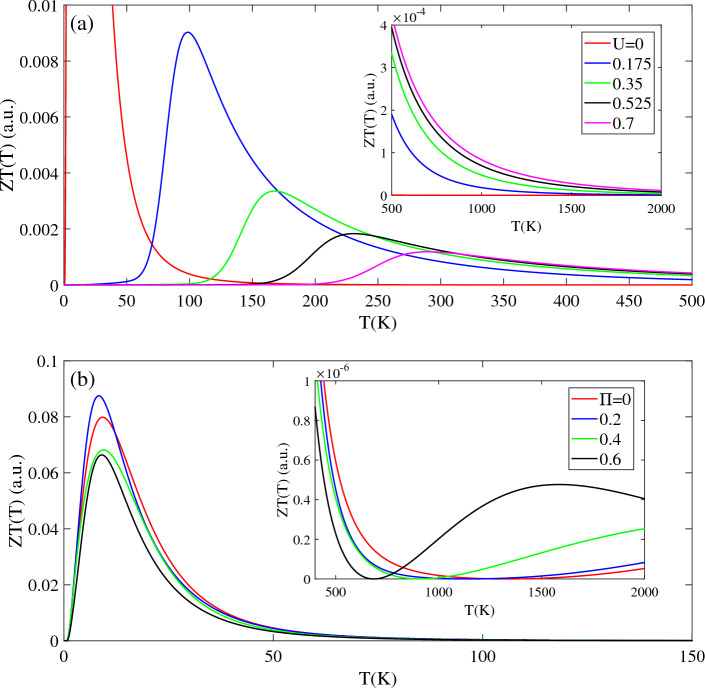
The figure of merit ZT(T) in terms of the temperature in the presence of the (**a**) bias voltage with Π = 0 and (**b**) magnetic field without bias voltage. Insets (**a**) and (**b**) show the ZT(T) in the higher temperature regions, respectively.

In Fig. 8, investigates the thermoelectric figure of merit ZT(T) as a function of temperature, with varying bias voltages, magnetic fields, and chemical potentials. ZT(T) is a crucial figure of merit that characterizes the thermoelectric performance of T-Ge. For the unbiased structure, ZT(T) exhibits a prominent peak with high intensity below 100 K, followed by a negligible intensity at higher temperatures (T > 1000 K), as indicated by the red line in Fig. 7a. ZT(T) is significantly influenced by the bias voltage. Compared to the unbiased structure, intensity of ZT(T) decreases in the presence of a bias voltage of U = 0.175 eV, with a peak appearing at 270 K. As the bias voltage increases to U = 0.375 eV and 0.525 eV, the peak of ZT(T) shifts to higher temperatures, around 510 K and 780 K, respectively, with a noticeable decrease in intensity. The strength of the bias voltage affected the ZT(T) throughout the temperature range 2000 K, which can be attributed to the opening and increasing of the band gap caused by the applied bias voltage. Above the 1000 K, the difference in intensity of ZT(T) with different bias voltage decreases, and the stronger bias voltage leads to a larger ZT(T).

As shown in Fig. 8b, investigation the temperature-dependent ZT(T) of unbiased T-Ge under different magnetic field strengths reveals several interesting trends in both the low [T < 150 K] and high [T < 2000 K] temperature regions. For all magnetic field strengths, a high-intensity peak is observed in ZT(T) at temperatures below 50 K, with approximately same intensity and position. Moreover, the behavior of ZT(T) of unbiased T-Ge in the low-temperature region (50 < T < 150 K) is not influenced by the Π strength, as indicated by the convergence of ZT(T) for all Π strengths towards a similar value. However, in the high-temperature region (T > 500 K), ZT(T) increases with increasing temperature, and stronger magnetic fields lead to higher intensities [inset of Fig. 8b]. Similar to the ZT(T) behavior of T-Ge at higher temperatures, the ZT(T) of monolayer group V graphyne exhibits an increasing trend rising temperature ^48^. Also, similar temperature-dependent increases in ZT(T) have also been reported for Silicene and Germanene^45,49^ and MoTe2^50^.

A comparing of the effects of magnetic field Π and bias voltage U on ZT(T) reveals significant differences, as depicted in Fig. 8a. While ZT(T) decreases with increasing U, its behavior remains unaffected by the strength of Π in the low-temperature region. However, in the high-temperature region, ZT(T) exhibits an increasing trend with Π.

**Figure 9:**
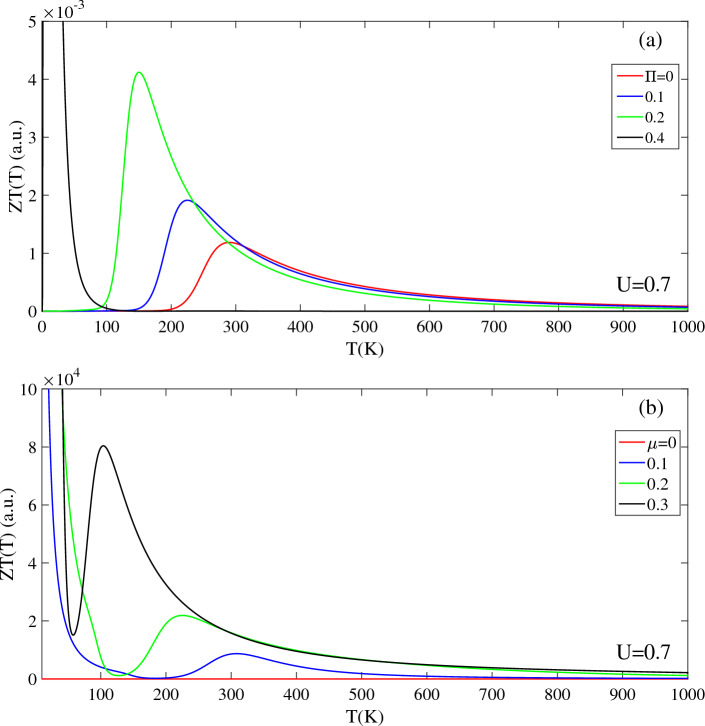
Effects of the (**a**) magnetic field and (**b**) chemical potential on the ZT(T) of biased T-Ge structure in terms of the temperature.

Figure 9 presents the effects of magnetic field and chemical potential on the ZT(T) of biased T-Ge with a bias voltage of U = 0.7 eV. In Fig. 9a, it is observed that in the absence of Π, the ZT(T, U = 0.7) is zero below a temperature of TZ = 200 K and exhibits a peak at T = 300 K, followed by a decreasing pattern at higher temperatures. When magnetic fields of Π = 0.1 and 0.2 are applied to ZT(T, U = 0.7), the ZT(U,Π) remains zero up to temperatures of 150 K and 100 K, respectively. Beyond these temperatures, the ZT(U, Π) shows an increasing trend until it reaches a peak at 210 K and 160 K, respectively. The magnetic field shifts the peak of ZT(U = 0.7,Π) to lower temperatures and enhances its intensity. For a stronger magnetic field of Π = 0.4, the intensity of ZT(U = 0.7,Π) significantly increases in the temperature range below 100 K. At temperatures above T > 300 K, the ZT(U = 0.7,Π) values are similar for all cases, and the intensity of ZT(U = 0.7,Π) decreases by reduction the magnetic field strength. Notably, as temperature approaches 1000 K, the ZT(U = 0.7,Π) values for all cases converge to the same value.

Figure 9b shows the effects of applying a chemical potential µ on the ZT(T, U = 0.7) of a biased T-Ge with U = 0.7 eV. The presence of μ greatly influences the intensity of ZT(T, U = 0.7) with µ ≠ 0 exhibiting a significantly higher intensity compared to µ = 0. Furthermore, for all selected values of µ, the ZT(T, U = 0.7) demonstrates a high intensity at temperatures below 100 K. When µ is set to 0.1 eV, the ZT(T, U = 0.7) reaches zero at approximately T = 200 K and then increases, reaching a peak value at approximately T = 300 K. Similarly, the ZT(T, U = 0.7) values for µ = 0.2 eV and 0.3 eV also exhibit similar trend, but with different intensities. Specifically, the peak of ZT(T, U = 0.7) shifts to T = 230 K for µ = 0.2 eV, while it shifts to T = 120 K for µ = 0.3 eV. Additionally, it is worth noting that larger values of µ exhibit higher intensities for the ZT(T, U = 0.7) below 400 K. At higher temperatures, around 1000 K, all selected values of µ exhibit nearly the same strength, with only negligible differences.

These findings highlight the significant influence of magnetic field and µ on the thermoelectric properties of biased T-Ge, particularly at low temperatures. These observations indicate that the ZT(T) of biased T-Ge is highly influenced by the temperature range and the strength of the applied magnetic field and chemical potential.

## Effects of the chemical potentially on thermal properties

**Figure 10:**
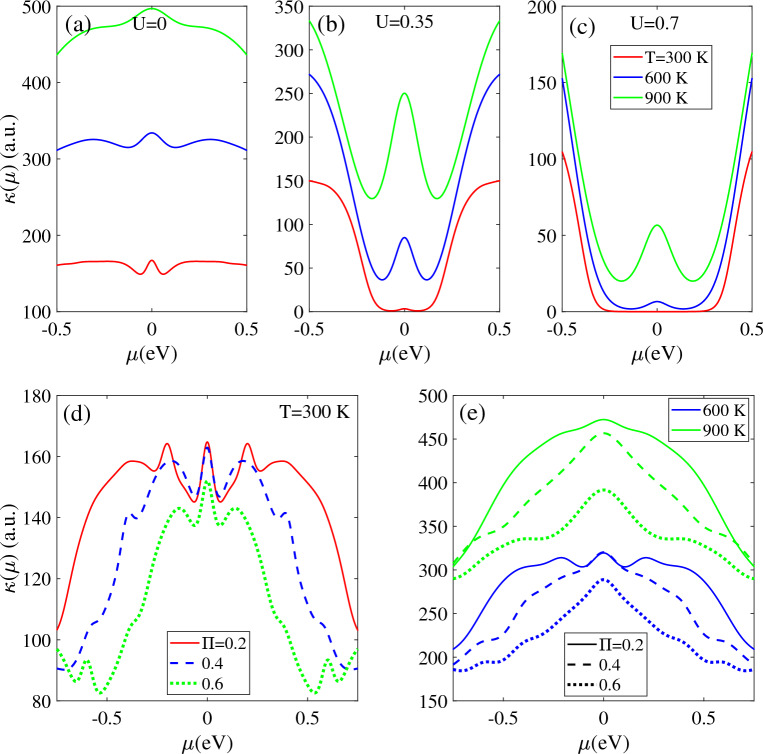
Thermal conductivity as a function of μ in the presence of (**a**)–(**c**) bias voltage with different temperatures T = 300 K, 600 K and 900 K, respectively. Thermal conductivity as a function of μ in the presence of different magnetic field strengths at (**d**) T = 300 K and (**e**) T = 600 and 900 K, respectively.

Figure 10 depicts the behavior of thermal conductivity as a function of the parameter µ at various temperatures (300 K, 600 K, and 900 K) for the biased T-Ge structure. The behavior of thermal conductivity is symmetric for n-type (µ > 0) and p-type (µ < 0) doping, owing to the symmetric nature of the electronic structure for valence and conduction bands.

For the unbiased T-Ge, the thermal conductivity κ(µ) remains relatively constant across different values of µ for all selected temperatures, except for around µ = 0. Moreover, it is observed that κ(µ) is larger for the higher temperature value [Fig. 10a].

The biased T-Ge structure exhibits lower thermal conductivity than the unbiased structure, and the reduction becomes more pronounced as the bias voltage increases. This is because the band gap increases with bias voltage, which decreases the density of charge carrier and leads to a lower thermal conductivity. At the T = 300 K, in the biased structure with U = 0.35 eV, the thermal conductivity is zero within the range |µ|< 0.12 eV due to the opening of the band gap. This range widens as the bias voltage increases to U = 0.7 eV, as indicated by the red lines in Fig. 10b and c. As the temperature increases to 600 K and 900 K, the thermal conductivity becomes non-zero across all values of µ and its intensity increases. The thermal conductivity is higher at T = 900 K compared to T = 300 K and 600 K, which can be attributed to the increase in thermal energy and a larger number of carriers available for transition processes. In agreement with biased T-Ge, monolayer InSe also exhibits a similar dependence of κ(μ) on μ^51^.

Figure 10c and d illustrate the impact of the magnetic field on the thermal conductivity of unbiased T-Ge at different temperature values of 300 K, 600 K, and 900 K. Across all cases, the thermal conductivity, κ(µ), has a maximum values at µ = 0 and decreases as µ changes. This behavior occurs because the unbiased T-Ge has a zero band gap and when the magnetic field increases, more charge carriers are excited to higher energy levels which in turn increases scattering and decreases their mobility. Notably, the thermal conductivity κ(µ) reaches its maximum value for a smaller magnetic field strength, regardless of the temperature value [Fig. 10c and d for Π = 0.2]. Conversely, the intensity of κ(µ) is the smallest for Π = 0.6 across all values of µ. It is worth mentioning that the thermal conductivity increases with temperature at a constant Π, with the highest values observed at T = 900 K.

**Figure 11:**
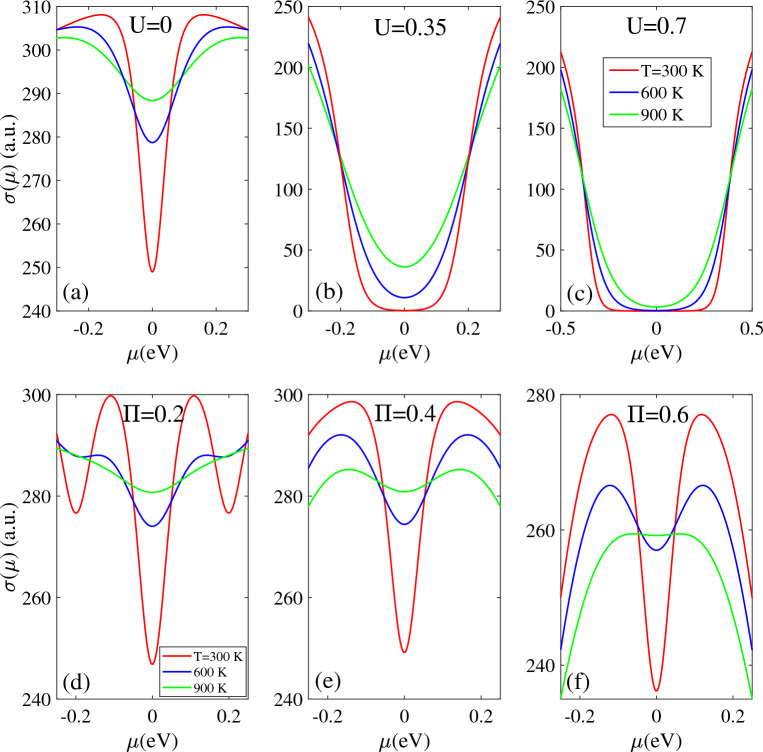
Electrical conductivity as a function of µ in the presence of (**a**)–(**c**) bias voltage and (**d**)–(**f**) magnetic field for the different temperatures T = 300 K (red), 600 K (blue) and 900 K (green).

Similar to thermal conductivity, the electrical conductivity is sensitive to doping parameter μ. As shown in Fig. 11, the bias voltage and magnetic field also influence its strength at different temperature values.

The temperature directly affects the electrical conductivity, with higher temperatures leading to larger values of σ(µ) across all values of µ. In the absence of external fields, the σ(µ) exhibits a minimum value at µ = 0 and increases as |µ| increases up to 0.16 eV, after which it decreases again. The enhancement temperature directly affects the σ(µ), with higher temperature T = 900 K leading to higher strength across the full range of µ. The results are in good agreement with previous DFT calculation^30^. When the bias voltage is applied, the σ(µ) remains close to zero in |µ|< 0.1 eV due to band gap opening caused by the bias voltage. For |μ| above this range, σ(μ) increases with further negative/positive increases in µ. When the bias voltage is increased to U = 0.7 eV, the σ(µ) remains zero for |µ|≤ 0.25 eV. Outside this range, as |µ| increases, the σ(µ) becomes non-zero and increases. As shown in Fig. 11b and c, beyond the zero region µ, higher temperature result in larger σ(µ) and this behavior continues until |µ| reaches ± 0.21 eV and ± 0.4 eV, where the trend is reversed. It can be concluded that electrical conductivity of biased T-Ge exhibits several notable behaviors including: (i) symmetric behavior of σ(µ) with respect to positive and negative µ values, (ii) increasing the intensity of σ(µ) with temperature in all µ region and (iii) increasing the zero intensity region for σ(µ) as the bias voltage increases.

Figure 11d, e and f show that the electrical conductivity of T-Ge is significantly affected by the application of a magnetic field. This is due to the distinct metallic properties of T-Ge at different Π values. At a temperature of 300 K and a Π value of 0.2, the σ(µ) exhibits two minimum values at µ = 0 and 0.2 eV, with a maximum value at µ = 0.1 eV [Fig. 11d]. However, when the magnetic field is raised to Π = 0.4 and 0.6, the σ(µ) remains at a minimum at µ = 0 and then reaches its maximum value at µ = 0.12 eV [Fig. 11e and f]. For each selected temperature, the intensity of σ(µ) is approximately the same for Π = 0.2 and 0.4, with negligible differences and both are greater than the intensity for Π = 0.6.

## Conclusions

This study investigates the thermoelectric properties of the 2D T-Ge, using the Kubo-Greenwood formula and a tight binding model with a Green function approach. The results show that the electrical/thermal conductivity, figure of merit, and power factor of T-Ge are significantly influenced by the bias voltage, magnetic field, and chemical potential. T-Ge exhibits a unique electronic structure with two Dirac points and a zero band gap, making it sensitive to external fields. We found that T-Ge remains metallic in the presence of a magnetic field, but the bias voltage opens and increases its band gap. In addition, the application of an external magnetic field leads to band splitting and the creation of additional band edge states, which can be attributed to the Zeeman Effects. Moreover, as temperature increases, the thermal and electrical conductivity of T-Ge also increase due to the increase in thermal energy of charge carriers. Modifying the band structure, particularly reducing the band gap, has a significant impact on the thermoelectric properties of T-Ge. The finding results demonstrate that the thermoelectric properties of T-Ge are highly dependent on the external parameters such as magnetic field, bias voltage and chemical potential. Specifically, we found that the thermoelectric properties of T-Ge decrease as the bias voltage increases, increase with the magnetic field due to the modification the band structure, and increase with the chemical potential due to the higher density of charge carriers. Tuning the band structure of T-Ge through external parameters can optimize its electrical conductivity, leading to an enhanced figure of merit (ZT), and improved thermoelectric performance. Controlling the thermoelectric properties of materials using bias and doping is crucial in industry as it can lead to the development of more efficient thermoelectric devices. The findings also demonstrate the potential of T-Ge for use in electronic and magnetic devices, opening up new possibilities for further research and development in this field.

## Supplementary Information

Supplementary Information.

## Data Availability

The datasets used and analyzed during the current study available from the corresponding author on reasonable request.
