# Supplementary material for: Tunable band gap and enhanced thermoelectric performance of tetragonal Germanene under bias voltage and chemical doping
Source: Sci Rep. 2023 Jul 25;13:12023. doi: 10.1038/s41598-023-39318-9 (PMC10368748; doi:10.1038/s41598-023-39318-9)
Supplement: Supplementary file 1 — Supplementary Information. [file 41598_2023_39318_MOESM1_ESM.pdf]

Supporting File

Tunable Band Gap and Enhanced Thermoelectric Performance of Tetragonal Germanene under Bias Voltage and Chemical Doping

Raad Chegel

[1]**Department of physics, Faculty of Science, Malayer University, Malayer, Iran**

E-mail address:raad.chegel@gmail.com

Calculation the thermoelectric based on the Kubo formula:

The calculation steps:

The effects of electric field (bias voltage), magnetic field, and impurity concentration on the temperature dependence of thermoelectric properties in doped T-Ge have been investigated using a tight binding model Hamiltonian.

Construction the Hamiltonian Matrix:

1. First, the real Hamiltonian matrix of system is constructed [Eq. 1.].
2. The Hamiltonian is obtained in the k-space based on the Fourier transformation for the creation and annihilation fermion operators [Eq. 3]. In this equation, the f_1 and f_2 are given based on the wave vector $\boldsymbol{k} = k_x\,\hat{e}_x + k_y\,\hat{e}_y$ in the first Brillouin zone.
3. In the presence of the bias voltage, the diagonal elements of the Hamiltonian included the bias strength.
4. By applying a magnetic field Π to the T-Ge structure, the overall Hamiltonian becomes spin-dependent and is represented by a 16×16 matrix.

Obtaining band structure: Effects of the bias voltage and magnetic field

5. The electronic band structure $E(\boldsymbol{k})$ can be obtained by solving the Schrödinger equation.
6. With an applied bias voltage, the Hamiltonian becomes an 8×8 matrix, resulting in 4 valence and 4 conduction subbands for biased T-Ge.
7. Under a magnetic field, the Hamiltonian becomes spin-dependent, leading to 8 valence subbands and 8 conduction subbands as the eigenvalues.

Density of States (DOS)

8. Using, the equation of motion for electron $[\sum_s[(E\boldsymbol{I} - \boldsymbol{\Omega}(\Pi))\delta_{is} + \boldsymbol{t}_{is}]\,\boldsymbol{G}_{sj}(E) = \boldsymbol{I}\delta_{ij}]$, the Green function matrix $[\boldsymbol{G}_{sj}(E)]$ has been obtained [Eq. 4].
9. Now, based on this equation, after Fourier transformation of Green function in the k-space, the density of state is obtained from $-\frac{\boldsymbol{1}}{\boldsymbol{\pi}}\boldsymbol{Im}[\boldsymbol{G}_{jj}(E)]$.

10. Calculation the thermoelectric properties required obtaining the spectral function $\boldsymbol{A}(\boldsymbol{k},\varepsilon) = -2Im\hat{G}(k,\varepsilon)$ which is associated with the density of states.

Thermal conductivity

11. According to the spectral function of the Green's function, the transport coefficients [$\Lambda_{qq'}$] is given by the Kubo relation [Eq. 10], as:

$$\Lambda_{qq'} = \frac{1}{\beta}\int_{-\infty}^{+\infty}\left[\frac{\partial f(\varepsilon)}{\partial\varepsilon}\right]\frac{d\varepsilon}{2\pi}\sum_{k,p}\Big(E_p(k,U,\Pi)\Big)^{q+q'-2}\left(\nu_k^{(p)}A_p(k,\varepsilon)\right)^2$$

$$\Lambda_{11} = \frac{1}{\beta}\int_{-\infty}^{+\infty}\left[\frac{\partial f(\varepsilon)}{\partial\varepsilon}\right]\frac{d\varepsilon}{2\pi}\sum_{k,p}\left(\nu_k^{(p)}A_p(k,\varepsilon)\right)^2$$

$$\Lambda_{12} = \frac{1}{\beta}\int_{-\infty}^{+\infty}\left[\frac{\partial f(\varepsilon)}{\partial\varepsilon}\right]\frac{d\varepsilon}{2\pi}\sum_{k,p}\Big(E_p(k,U,\Pi)\Big)^{1}\left(\nu_k^{(p)}A_p(k,\varepsilon)\right)^2$$

$$\Lambda_{22} = \frac{1}{\beta}\int_{-\infty}^{+\infty}\left[\frac{\partial f(\varepsilon)}{\partial\varepsilon}\right]\frac{d\varepsilon}{2\pi}\sum_{k,p}\Big(E_p(k,U,\Pi)\Big)^{2}\left(\nu_k^{(p)}A_p(k,\varepsilon)\right)^2$$

12. The electrical conductivity is proportional to transport coefficient Λ_{11}.
13. The thermal conductivity is obtained based on the transport coefficients, as [Eq. 11]:

$$\kappa(T) = \frac{k_B^2}{T}\left[\Lambda_{22}(T) - \frac{\Lambda_{12}(T)\Lambda_{21}(T)}{\Lambda_{11}(T)}\right]$$

Thermoelectric properties

14. Here, transport coefficients have been used to obtain the figure of merit ZT(T) and power factor PF(T). These thermoelectric coefficients are obtained based on the transport coefficients [$\Lambda_{qq'}$], as:

$$ZT(\mathrm{T}) = \frac{[\Lambda_{12}]^2}{\Lambda_{11}\Lambda_{22} - [\Lambda_{12}]^2}$$

$$\mathrm{PF}(T) = \frac{[\Lambda_{12}]^2}{\mathrm{T}^2\Lambda_{11}}$$

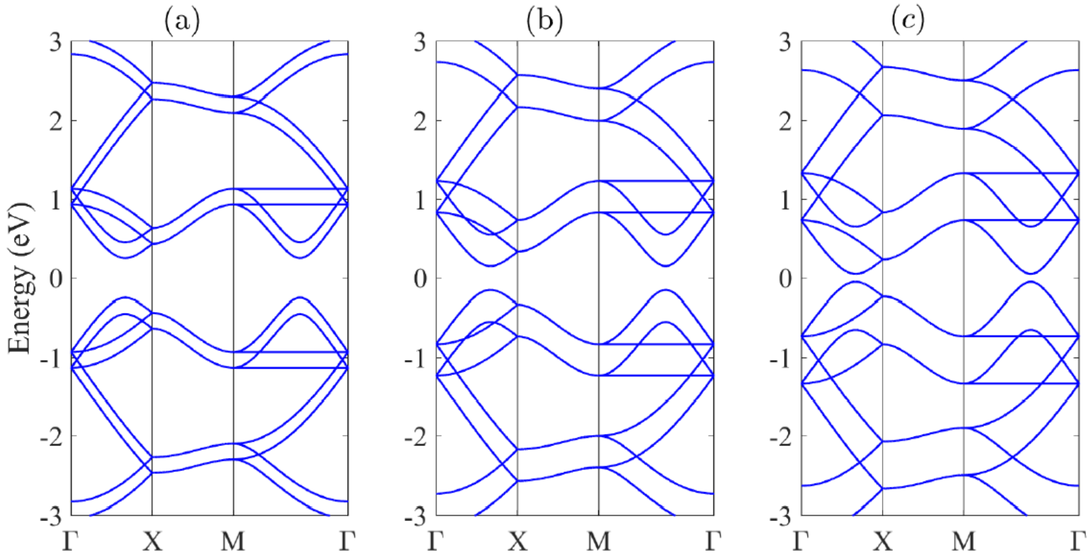

Figure S1. The band structure of the T-Ge with bias voltage U=0.7 eV in the presence of the magnetic fields (a)-(c) Π=0.1, 0.2 and 0.3, respectively. It can be found that, the non-zero band gap of biased T-Ge with U=0.7 eV decreases with magnetic field until it vanishes at a critical value of Π=0.35.

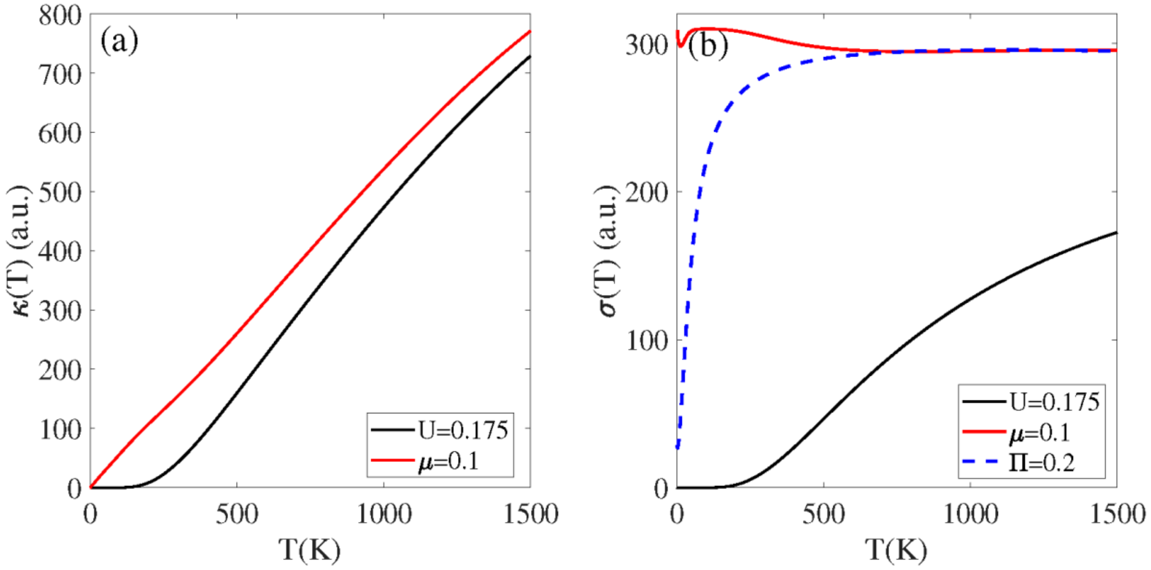

Figure S2. The (a) thermal conductivity and (b) electrical conductivity, in the presence of the bias voltage, magnetic field and chemical potential, respectively.
